# Understanding of Flower Initiation Pattern and Developmental Stages of Epiphyte orchid *Aerides odorata* Lour., from Sabah

**DOI:** 10.21315/tlsr2026.37.1.7

**Published:** 2026-03-31

**Authors:** Nor Amirah Shamsudin, Jualang Azlan Gansau, Ahmad Asnawi Mus, Heira Vanessa Nelson, Devina David, Nor Azizun Rusdi

**Affiliations:** 1 Institute for Tropical Biology and Conservation, Universiti Malaysia Sabah, Jalan UMS, 88400 Kota Kinabalu, Sabah, Malaysia; 2 Faculty of Science and Technology, Universiti Malaysia Sabah, Jalan UMS, 88400 Kota Kinabalu, Sabah, Malaysia; 3 Institute of Biodiversity and Environmental Conservation, Universiti Malaysia Sarawak, 94300 Kota Samarahan, Sarawak, Malaysia; 4 Faculty of Sustainable Agriculture, Universiti Malaysia Sabah, 90509 Sandakan, Sabah, Malaysia

**Keywords:** Capsule, Floral Development, Inflorescence, Spike, Senescence, Kapsul, Perkembangan Bunga, Infloresen, Tangkai (bunga), Penuaan

## Abstract

*Aerides odorata* Lour. is an epiphytic orchid with commercial potential due to its intriguing flowers. Nevertheless, the flowering patterns of this species have not been investigated. Flower initiation and development are important for plant reproduction because they mark the transition from vegetative growth to reproductive growth, allowing plants to produce flowers and ultimately, seeds or fruits. Besides, floral initiation and development in orchids differ among various species. This work seeks to observe and document the flowering stages and find the optimal maturity stage for *Aerides odorata* Lour. seed pod, which is crucial for *in vitro* asymbiotic seed germination. Three orchid plants with 2–3 peduncles each were observed daily over five months, every morning (9:00 a.m.–11:00 a.m.) to record development from spike initiation to full bloom, and subsequently for three months post-pollination until capsule dehiscence. Summarising the information on floral developmental stages involved four main phases: spike formation (considered the first day of the phases), bud initiation, flowering and senescence until seed pod formation. The results showed that approximately twelve significant floral developmental stages were systematically documented, each correlated with time intervals and morphological landmarks. Upon observation, it was noted that each of these species could create a maximum of 22 flower buds per plant. The flower is fully wilted 85 days after being pollinated. In addition, the pollinated flowers transformed into capsules and fully reached maturity after 100 days following pollination. Gaining insight into the floral initiation process in orchid species is crucial for optimising cultivation conditions and promoting successful flowering in these aesthetically pleasing and varied plants.

## Abstrak

Aerides odorata Lour. ialah orkid epifit yang mempunyai potensi komersial disebabkan bunganya yang menarik. Namun begitu, corak pembungaan spesies ini masih belum dikaji. Inisiasi dan perkembangan bunga adalah penting untuk pembiakan tumbuhan kerana ia menandakan peralihan daripada pertumbuhan vegetatif kepada pertumbuhan reproduktif, yang membolehkan tumbuhan menghasilkan bunga dan seterusnya biji benih atau buah. Selain itu, inisiasi dan perkembangan bunga dalam orkid berbeza antara spesies. Kajian ini bertujuan untuk memerhati dan mendokumentasikan peringkat pembungaan serta menentukan tahap kematangan optimum bagi kapsul biji Aerides odorata Lour., yang penting untuk percambahan biji secara asimbiotik in vitro. Tiga pokok orkid dengan 2–3 pedunkel setiap satu diperhatikan setiap hari selama lima bulan, setiap pagi (9:00 pagi–11:00 pagi), bagi merekodkan perkembangan daripada permulaan pembentukan tangkai bunga hingga ke peringkat bunga berkembang penuh. Pemerhatian diteruskan selama tiga bulan selepas pendebungaan sehingga kapsul merekah. Ringkasan maklumat mengenai peringkat perkembangan bunga melibatkan empat fasa utama iaitu pembentukan tangkai bunga (dianggap sebagai hari pertama fasa), inisiasi kudup, pembungaan, dan penuaan sehingga pembentukan kapsul biji. Hasil kajian menunjukkan bahawa kira-kira dua belas peringkat perkembangan bunga yang signifikan telah didokumentasikan secara sistematik, masing-masing berkait dengan sela masa dan penanda morfologi tertentu. Pemerhatian juga mendapati bahawa setiap pokok mampu menghasilkan maksimum 22 kudup bunga. Bunga akan layu sepenuhnya 85 hari selepas pendebungaan. Selain itu, bunga yang telah didebungakan akan berubah menjadi kapsul dan mencapai kematangan penuh selepas 100 hari pendebungaan. Pemahaman tentang proses inisiasi bunga dalam spesies orkid adalah penting untuk mengoptimumkan keadaan penanaman serta menggalakkan pembungaan yang berjaya dalam tumbuhan yang mempunyai nilai estetika dan kepelbagaian ini.

**HIGHLIGHTS**

- Twelve (12) distinct development stages from spike initiation to capsule dehiscence were systematically characterised.
- Each plant produced up to 22 flower buds, with unpollinated flowers lasting around 14 days before senescence.
- Optimal pollination (day 4–5) and capsule maturity (100 days–110 days) were identified.

## INTRODUCTION

**FIGURE 1:**
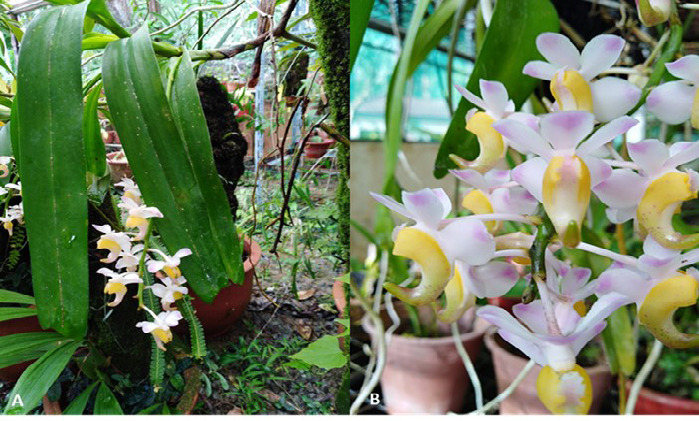
The flower of *Aerides odorata* Lour. (A) Whole plant view with flowers, (B) Close-up view of the flowers.

*Aerides odorata* is a fragrant orchid species widely distributed in Southeast Asia, Indonesia, the Philippines and other regions. It has been reported to show many differences, and these variations are even more noticeable across the species’ entire distribution in Peninsular Malaysia (Teoh 2021). In Indonesia, *A. odorata* is renowned and attracts flower collectors due to its distinctive colour and fragrance. The plant produces pendulous sprays of waxy, very fragrant, pale pink flowers with a touch of deeper pink in the lip, and is known for its highly fragrant, cinnamon, clove or lilac-scented flowers. It blooms in late spring through fall on a long, many-flowered inflorescence from the leaf axils (Paraste *et al*. 2023). Although its medicinal properties have been acknowledged since ancient times, it has not attained the same level of popularity as its potential as an ornamental plant (Sulistiarini 2008). This has resulted in excessive collection from its natural habitat on a large scale. In the Convention on International Trade in Endangered Species of Wild Fauna and Flora (CITES), this species is listed under Appendix II, which means this species can be traded. Still, it is subject to regulations and limited in quantity (Khalida *et al*. 2019). According to Teoh (2021), the floral specimen used in this study is conclusively recognised as *Aerides odorata* Lour. In contrast, Sivanaswari *et al*. (2011) identified this specimen as *Aerides odorata* var. ‘yellow’ based on the prominent yellow colouring observed on its spur (Fig. 1).

Fig. 1 shows the floral morphology of *Aerides odorata* Lour. at different observational scales.

Orchids are fascinating because of their distinctive flowering patterns and notable variances in reproductive organs, which display considerable physical differences (Balilashaki *et al*. 2015). Floral initiation and development are essential processes in the life cycle of orchids, including *A. odorata*. It helps us understand how flower parts form in plants and connects to various areas of plant biology, like systematics, developmental genetics, physiology, biophysics and many more (Yoshida *et al*. 2021, Iwamoto & Bull-Hereñu 2018). The study on flower initiation, morphology and development of flowers and fruits is essential to determine the factors limiting seed production. Identifying the optimal timing for flower induction is crucial for enhancing fruiting, making the time of flower initiation a significant aspect of this sequence (Bakar *et al*. 2014). Studying the development cycle from flowering to fruiting can serve as a valuable guide to accurately predict the best time for harvesting, leading to improved efficiency (Guo *et al*. 2022; Mohamad & Rusdi 2020; Nelson *et al*. 2024). By keeping track of the number of flowers, fruits and seeds produced during this cycle, we can determine the plant’s reproductive potential and estimate its ability to produce seeds effectively (Syamsuwida *et al*. 2012).

Despite the horticultural and ecological importance of *A. odorata*, there remains a lack of research on its specific patterns of flower initiation and significant events related to seed development. This gap raised an important question: Does the sequence of flower initiation, growth and development in orchids culminate in capsule formation vary among orchid species compared to other flowering plants? Accordingly, the present study aims to provide a baseline framework describing the floral development of *A. odorata*. Previous research has focused mainly on pollination biology, seed germination or reproductive traits (Nilsson 1992; Wang *et al*., 2019; Li *et al*. 2022) without giving an explicit morphological characterisation of the developmental stage.

We hypothesise that this species undergoes a distinct and sequential series of floral initiation and capsule development stages, which can be systematically documented and correlated with specific time intervals. Observations on the developmental cycle from flowering to fruiting may serve as a valuable guide for predicting the optimal harvesting time, thereby improving efficiency. The species’ reproductive potential can be assessed by quantifying the number of flowers and capsules or pods produced throughout the cycle, allowing for a more accurate estimation of capsule or pod production capacity. Furthermore, this knowledge provides essential information for determining the precise timing of hand pollination in orchid seed, as it is directly informed by an understanding of floral structures and their pollination characteristics.

The study aimed to characterise the floral morphology and initiation of *A. odorata*, and to document the sequential stages of flowering and fruiting development, including assessing its reproductive success during the flowering period. This is the first comprehensive detailing the complete sequence of flowering and capsule development of *A. odorata* from Sabah, Borneo. No study has provided a detailed stage-by-stage morphological framework of the whole sequence of flowering and capsule development in *A. odorata*. Establishing these stages will not only strengthen our understanding of the reproductive biology of *A. odorata* but also provide critical guidance for conservation and *in vitro* propagation practices

## MATERIAL AND METHODS

### Plant Material

A total of five plants of *A. odorata* were collected from Kg. Lobong-Lobong, Kota Belud, Sabah (05°03′93″ N, 116°43′72″ E), elevation at 1,200 m and kept in the greenhouse under natural light, 80% relative humidity, with a temperature of 28.24 ± 2°C day/night located at the Institute for Tropical Biology and Conservation, Universiti Malaysia Sabah (06°03′33″ N, 116°012′29″E and at an elevation at 110 m. These orchids were also repotted with sphagnum moss and coconut husk medium and fertilised using commercial fertiliser (FT Grow), which was supplied once every three weeks to promote good growth and induce flowering. All the flowers were watered twice daily and placed under natural light conditions with 80% humidity at an average temperature of 24°C/28°C (night and day). Three healthy orchid plants, each with 2–3 peduncles, were selected for observation during the observation period. Although the limited number of plants represents a constraint of this study, the presence of several peduncles per plant provided multiple observational units, thereby increasing the robustness of the data.

### Observation of Flower Initiation and Formation

The observation and monitoring were carried out daily in the morning from 9:00 a.m. to 11:00 a.m. for five consecutive months, according to Mohamad and Rusdi (2020). The plant’s longevity was observed from the formation of the spike till the end of flower senescence. Observation was conducted in two phases:

1. Intensive daily monitoring of spike initiation throughout the flowering phase over two months.
2. Subsequent monitoring of capsule development progression up to three months post-pollination, with systematic weekly observations documented during this phase.

The size of the spike, mature flower bud, flower (from dorsal to lateral sepal), number of flowers produced per plant, length of inflorescence and seed capsule were observed using a ruler and Vernier calliper. The length of a flower bud is determined by measuring the distance from its base to its tip. Meanwhile, the width or diameter of a flower bud refers to the measurement taken across the broadest section of the bud. Since there is still no information on the flower’s lifespan, two flowers were left undisturbed on each orchid plant to track their longevity and morphological changes.

### Hand Pollination Technique

The present study employed a hand pollination method following the procedure previously reported by Kauth *et al*. (2008) and Nelson *et al*. (2024) with slight adjustments. Pollination occurred four to five days after the flowers had fully bloomed, and the process was carried out in the early morning, when the flowers were fresher. The initial step involved identifying fully open flowers and focusing on the columns and other caps. Subsequently, the anther caps and pollinia were delicately removed from the gynostemium with a pin. A gentle upward pressure was applied to the base of the anther caps, allowing them to adhere to the toothpick upon contact. Next, the anther caps were cautiously removed, and the pollens were carefully deposited onto the stigmatic surfaces of the same or another flower. This process was repeated several times to maximise pollen transfer. Upon the completion of hand pollination, the capsules were labelled with the parent plants’ information and the date of pollination. Subsequently, the flower senescence, capsule development and capsule dehiscence were observed.

### Flowering Morphology and Capsule Development

Developmental stages such as flower initiation, floral bud development, flower structure and morphology, and capsule development were examined in individual inflorescences (from fruit initiation until maturation). Time (day and period), shape, and colour were used to record changes in flower bud structure and blossoming (Owens *et al*. 1996). Throughout the developmental stage observation, the number of flowers and capsule fruit per inflorescence was recorded to evaluate the plant’s reproductive success.

For capsule development, the changes after pollination until the formation of the capsule were observed daily. The diameter and length of the capsule are also recorded and taken using a vernier calliper. The mature seed capsule is harvested before dehisced (on 110 days) (Balilashaki *et al*. 2015).

## RESULTS AND DISCUSSION

### Description of Flower Initiation Development Event

**FIGURE 2:**
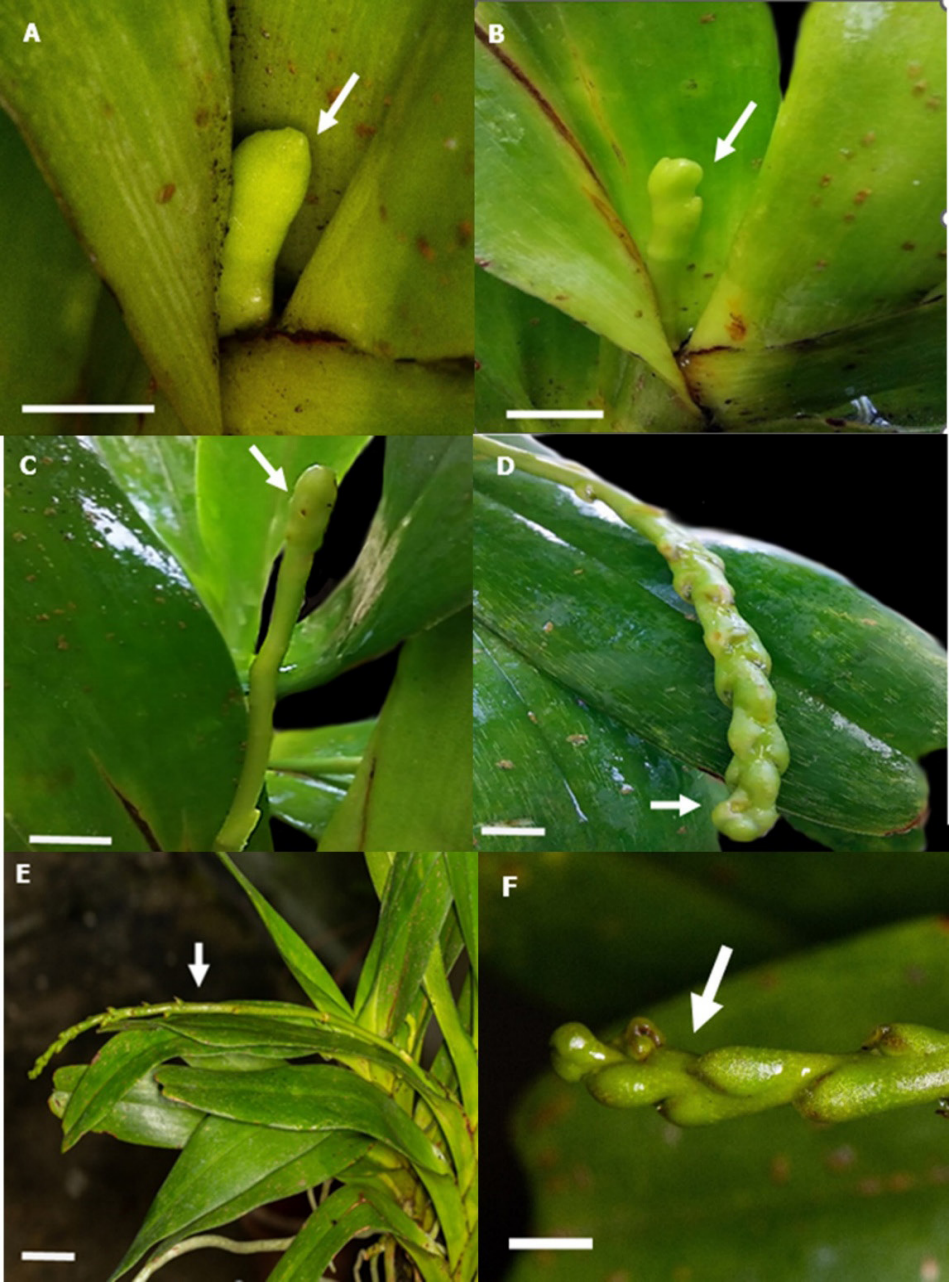
The stage of initiation and development of the spike and bud of *A. odorata*. (A) The emergence of the spike (Arrow). (B) The swelling of the spike on the 6th day (Arrow). (C) The elongation of the swollen spike (Arrow). (D) The appearance of a floral bud on the 20th day. (E) The elongation of the peduncle and flower stalk on the 37th day. (F) Close view of the floral bud (Bar = 1 cm, A–F).

**FIGURE 3:**
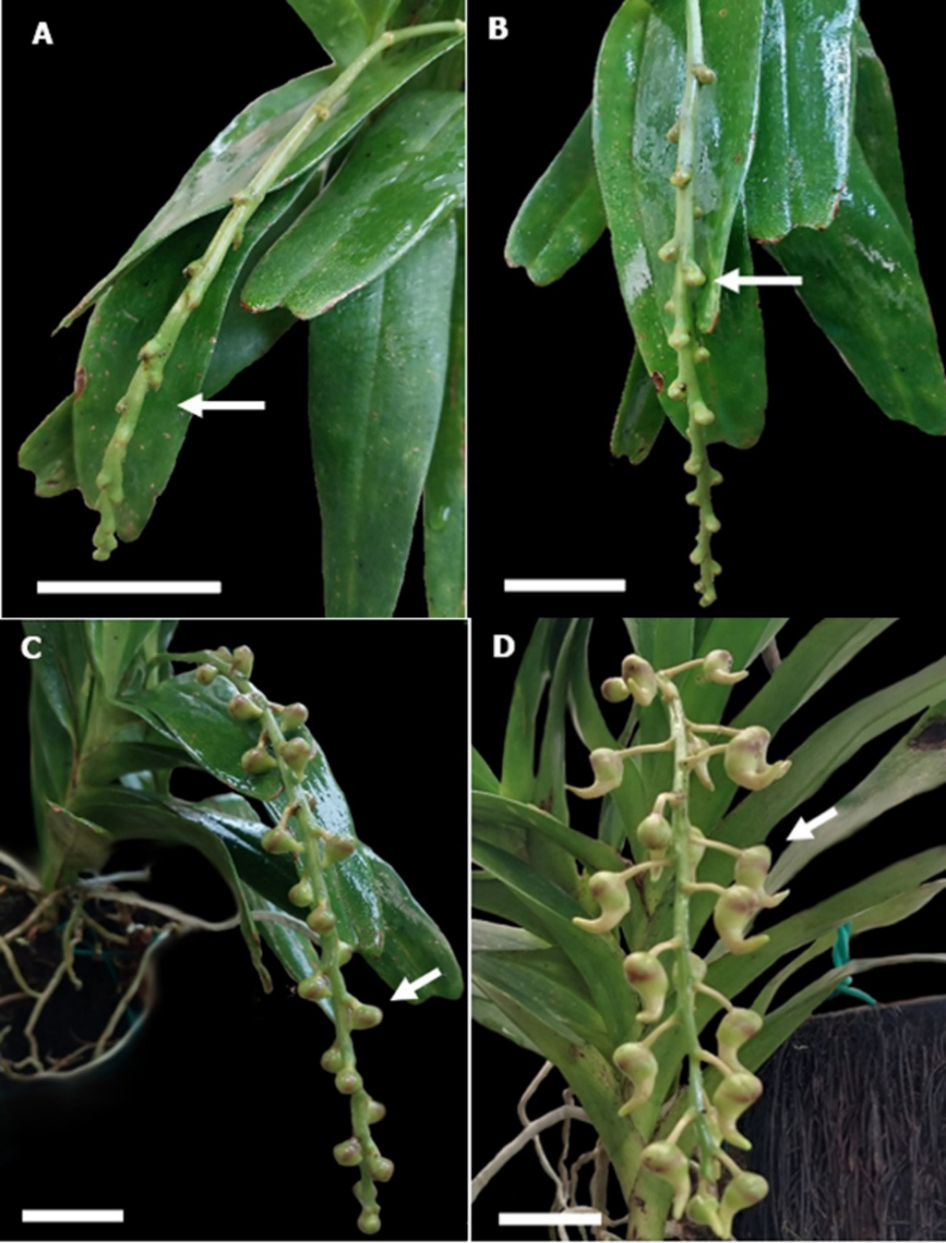
Floral development of *A. odorata*. (A) Peduncle elongation which holds the floral bud on the 37th day (Arrow). (B) Floral bud appearance on the 41st day (arrow). (C) The peduncle and pedicle of flow appear longer and partially matured bud on the 44th day (Arrow). (D) Mature flower bud–52nd day (Arrow) (Bar = 1 cm, A–D).

The flower developmental stages of *A. odorata* can be studied through 12 significant events, beginning with the initial phases (Stages 1–5), characterised as periods of growth during which the bud forms and develops into a bloom. The details of each developmental stage are now thoroughly outlined in Table 1, which summarises the corresponding durations and key morphological characteristics ranging from the initial spike swelling (Stage 1) to capsule dehiscence (Stage 12). The progression and sequence of events were consistent and uniform across all observed inflorescences in each plant studied. Illustrative images in Figs. 2, 3 and 7 highlight the significant transitions between stages. The floral initiation process for *A. odorata* commenced in mid-October and extended until the conclusion of November, during which buds emerged. By early December, the plant had fully bloomed. At this stage, the complete formation of a bud typically requires approximately 14 days. As a bud matures, it changes colour, transitioning from dark green to vibrant pink. The maturation phase of the cycle commences during stages 5–7, marked by the emergence of developed buds that subsequently develop into a fully bloomed flower with intact petals and sepals (Table 1). Finally, the senescence stages are stages 7 to 9. Once the flower of *A. odorata* has reached full bloom, it will begin to wither and lose its vitality within 14 days. The tip of the flower withered first and then turned brown. After 23 days, the pollinated flowers transformed into capsules. Finally, a fully developed mature capsule after 100 days following pollination. The comprehensive floral growth process and the length required for each stage can be succinctly summarised in Table 1.

#### Spike initiation and flower bud formation phase

Based on the observation, the floral spike of this species appears on the fourth to sixth node below the apical leaf. The spike initiation for *A. odorata* occurred in early October, signifying the onset of the first flowering stage for this plant. The initial size of the spike was approximately 0.60 ± 0.20 cm on average. The spike exhibits a pale green, as shown in Fig. 2A. Following five to seven days, the spike began to enlarge and exhibited a colouration that may be described as a combination of light green and yellow, as visible in Fig. 2B. This stage signifies the emergence of floral buds. The enlarged spike persists for four days before extending and developing into a floral bud at the peduncle of the stem. During the early phases of the flowering process, the initial spike undergoes growth. Subsequently, it emerges from the leaf base (Fig. 2C). Following that, the bud experiences elongation, forming a flowering spike that typically takes approximately 3 to 4 weeks. The length of the flower bud and peduncle steadily increased until it reached the bud initiation phase. The basal bud will initiate elongation before any other bud and develop into a distinct individual bud by the 20th day (Fig. 2D).

After 20 days, the spike started swell and steadily increased in size, producing flower buds. The spike initiation process commences once the spike reaches a length of 8.68 ± 1.55 cm. Observations are conducted on the early primordial growth until the bud reaches maturity, which is indicated by a bit of flower bud opening. The flower bud, also known as the multiplication of a floral bud, emerges 32 days after the spike swells (Fig. 2E). The flower bud undergoes simultaneous elongation and development, resulting in the production of a distinct bud structure (Figs. 2E and 2F) and a flower stalk known as a pedicle. The floral peduncle, which supports the pedicle, is 29.86 ± 1.12 cm long and ceases growth as the flower opens.

#### The flower bud development and maturation phase

Following three days of bud swelling, the flower peduncle undergoes rapid development and elongation. Simultaneously, it undergoes differentiation, developing into a compact spherical floral bud before forming a pedicel. Upon observing three distinct plant species, it was noted that each flowering plant could create a maximum of 2–3 peduncles with 22 flowers. Fig. 3A illustrates the development of a floral bud beneath the bract. After a few days, a rounded flower bud shape emerges from the bract (Fig. 3B). Additionally, the shape of the bud changes as it begins to form the first structure of a straight spur (Fig. 3C). The apex of the flower bud exhibits a purple ring hue, accompanied by a pale green shade on the spur. The fully developed flower bud becomes visible after 52 days. At this stage, the individual structures of the flower begin to grow within the bud, and the spur becomes more indented (Fig. 3D).

**FIGURE 4:**
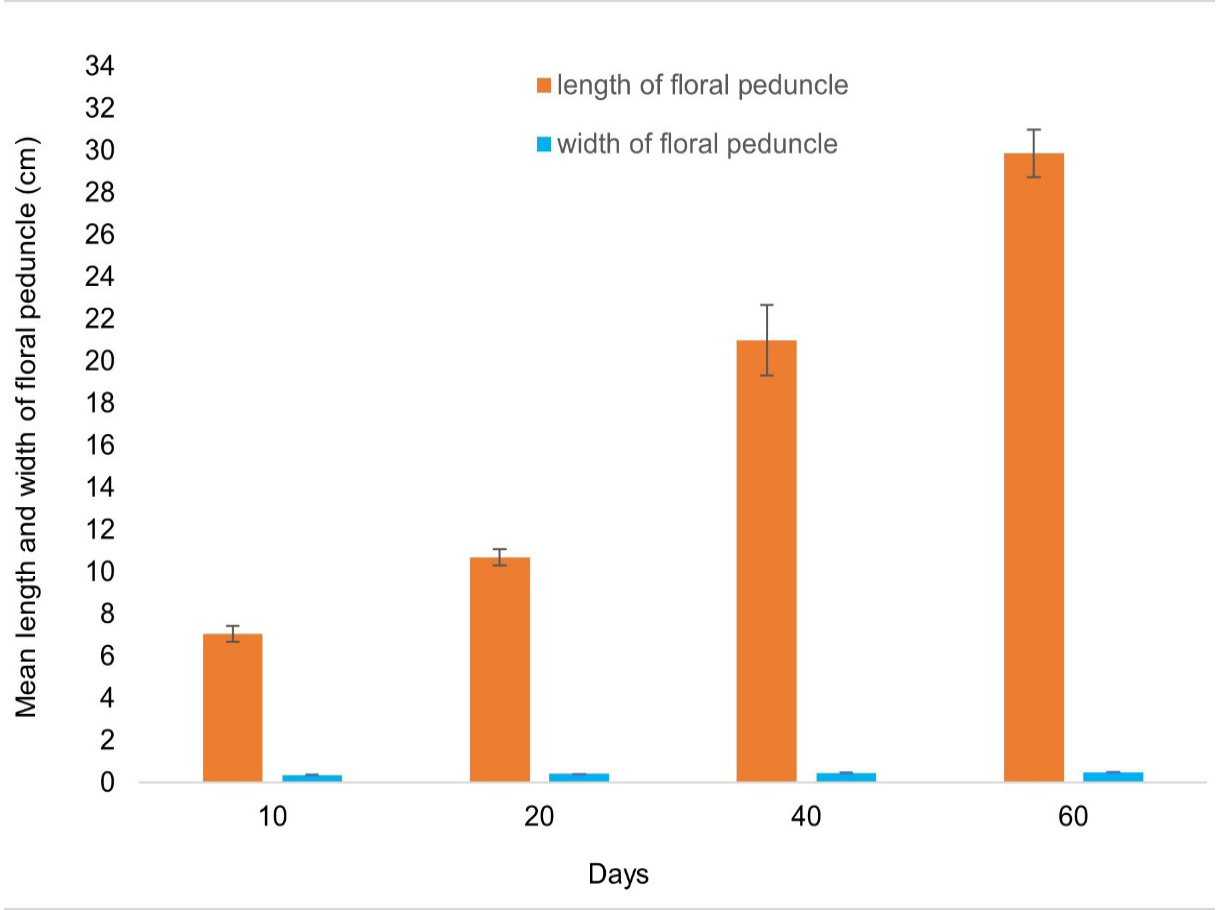
Mean length and width of floral peduncle for *A. odorata* at intervals 10 days, 20 days, 40 days and 60 days of floral bud development.

**FIGURE 5:**
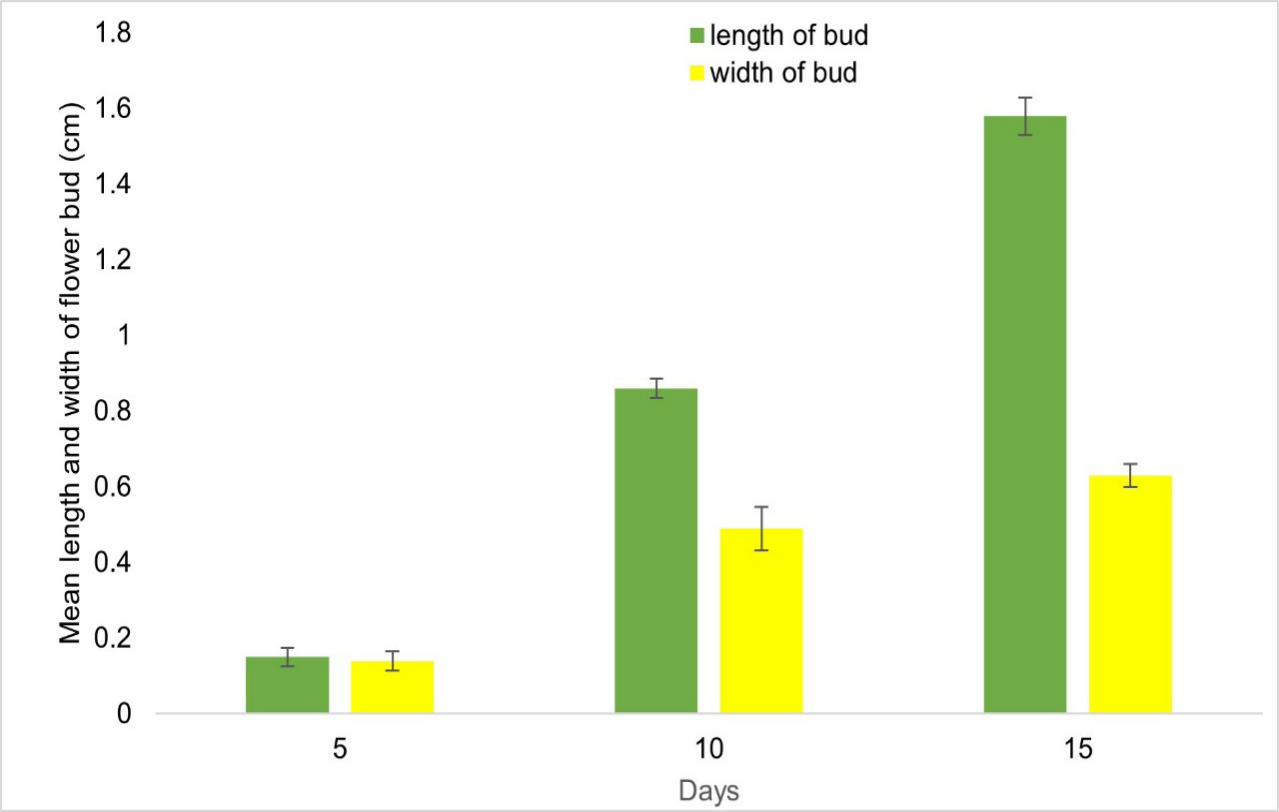
Mean length and width of flower bud for *A. odorata* at an interval of 5 days, 10 days and 15 days of floral bud development.

The average length and width of the floral peduncle from when it starts to form individual flower buds until 20 days later are shown in Fig. 4. The floral peduncle is the stalk or stem that supports a flower or a cluster of flowers. It has a vital function in offering structural reinforcement and facilitating the distribution of nutrients to the developing flowers. The dimensions of a floral peduncle can exhibit variation among plant species and even within the same plant’s distinct flowers. The length and width of *A. odorata* constantly increase throughout 20 days of observations. The flower bud’s length and width exhibited a consistent growth pattern, similar to that of floral peduncles (Fig. 5).

### The Development and Maturation Stages of the Flower

**FIGURE 6:**
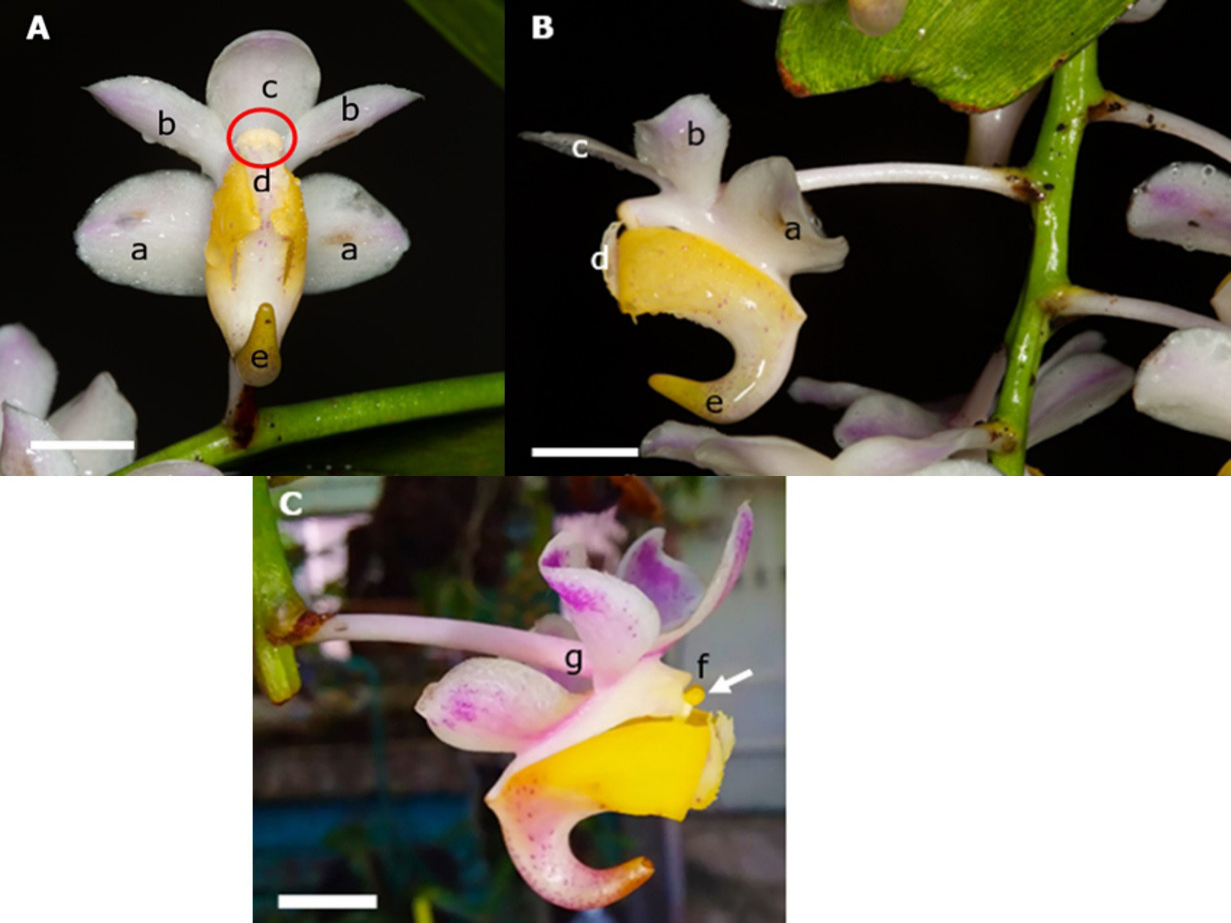
Flower morphology. (A) Front view of *A. odorata* flower – location of anther cap and pollinia (red circle). (B) Side view of *A. odorata* flower. a = lateral sepal; b = petal; c = dorsal sepal; d = labellum/lip; e = spur; f = pollinia (arrow); g = ovary (Bar = 1 cm).

The growth and maturity phases of the *A. odorata* flower are documented using an undisturbed and unpollinated orchid plant. The blooming of *A. odorata* in this investigation was observed to occur from November to December. The flowering period of *A. odorata* varies depending on its natural habitat. *A. odorata* exhibits prolific flowering, with 9 to 15 flowers clustered in a structure known as racemes. A raceme is a floral arrangement characterised by each flower’s growth from the leaf’s axil on an elongated, unbranched stalk (Figs. 6A and 6B).

The flower of *A. odorata* started to open on day 57 and fully bloomed 10 days after that (Fig. 6C). It took about 85 days for *A. odorata* to complete its floral development from the spike formation to the dehiscence of its flower. The flower will exhibit a dehiscence sign, characterised by the wilting of the sepal and petal, which will cause each end of the structure to turn brown, followed by the spur. Although “flower senescence” is often used, it rarely refers to the flower. Instead, it is more specifically used to describe the perianth sections of the flower.

### The Capsule Formation and Development

**FIGURE 7:**
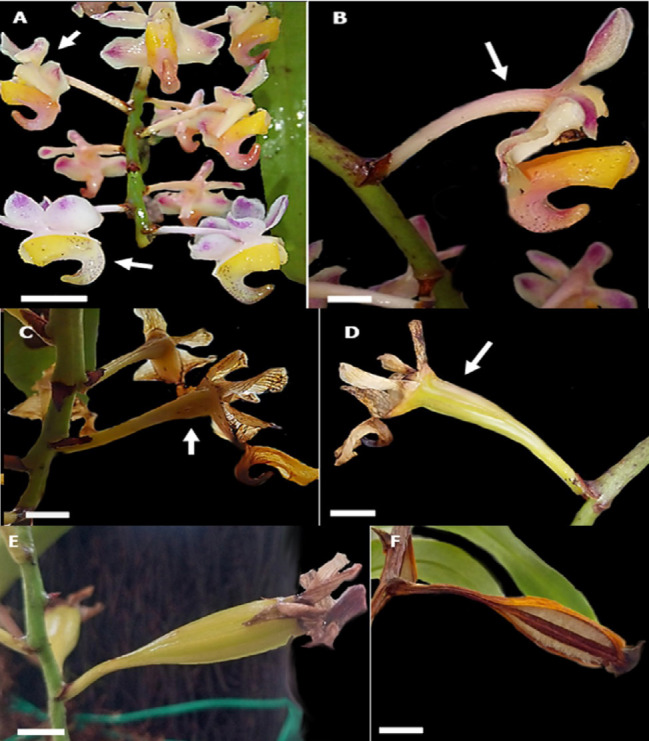
(A) The flower started the colour change 3 days after pollination (DAP) compared to unpollinated the flower; (B) The ovary swelled, and diameter increased (arrow); (C) Formation of the immature capsule 7 DAP; (D) Capsule increase in size 14 DAP; (E) Capsule 27 DAP; (F) capsule are dehiscence at 109 DAP (Bar = 1 cm, A–F).

In this part, the capsule formation was pollinated through artificial or hand pollination. Some mature flowers were hand-pollinated to induce capsule (pod) development, while others were left unpollinated to monitor the natural process of floral senescence. The colour of the *A. odorata* flower begins to alter three days after pollination occurs (Fig. 7A). The lip colour of *A. odorata* darkened as the concentration of anthocyanin increased, resulting in the wilting, and shrinking of the perianth (Fig. 7B). The ovary structure gradually increases in size and begins to transform into a seed capsule (Figs. 7C, 7D and 7E) that open through 3 to 4 longitudinal slits. Still, the ends of the structure remain closed together (Fig. 7F). The findings regarding the flowering duration in this species indicate that unpollinated flowers of *A. odorata* stay fresh and in bloom for 2 weeks before they begin to wilt. In contrast, pollinated flowers undergo senescence 5 days after pollination, suggesting a rapid onset of senescence-related processes triggered by pollination.

Out of all the seed capsule ages, the seed capsule that is 100 days old has the most considerable length, measuring 2.57 ± 0.11 cm. This is followed by the 90-day-old seed capsule, which measures 2.41 ± 0.03 cm, and the 60-day-old seed capsule, which measures 1.87 ± 0.016 cm. Regarding the breadth of the seed capsules, the 90-day seed capsule has the greatest width, measuring 0.69 ± 0.04 cm. It is followed by the 100-day seed capsule, which has a diameter of 0.52 ± 0.06 cm, and the 60-day seed capsule, which has a width of 0.36 ± 0.03 cm. According to the categories, the seed capsule, the seed capsule of *A. odorata* is considered a small seed capsule with a length of 2.57 ± 0.11 cm.

## DISCUSSION

Floral initiation in orchid species is a pivotal developmental event that signifies the shift from vegetative growth to the reproductive phase, ultimately forming orchid flowers. After floral initiation, orchids progress through a sequence of developmental stages, which involve the creation of flower buds, the specialisation of floral organs and ultimately the blossoming of the flowers. Orchid blooms exhibit great specialisation and display various forms, colours and sizes. The blooming season of *A. odarata* varies throughout different countries. In China, it occurs in May (Chen & Wood 2009), in Bhutan, it appears from March to June (Gurung 2006), and in Singapore, it happens from August to September (Teoh 2016).

Flower developmental stages can vary significantly among flowering plants due to differences in genetics, hormonal regulation, life cycles and environmental adaptations (Singh *et al*. 2023). Nevertheless, the basic stages of flower development are generally conserved, comprising floral induction, floral meristem formation, floral organ formation and maturation. Based on our observations, it has been documented that there is a total of 12 phases in the development of *A*. *odorata* flowers.

The distinctive contribution of this study is establishing, for the first time, a comprehensive sequence of 12 developmental stages for *A. odorata* from Sabah. This detailed framework advances the understanding of orchid floral biology by providing a valuable reference for identifying optimal pollination periods and determining capsule collection times for *in vitro* propagation. This result parallels those of *Paraphaleonopsis labukensis*, which also exhibited 12 primary developmental stages, although the duration of each stage differed (Nelson *et al*. 2023). In contrast, research on *Renanthera bella* documented only 10 landmark events (Mohamad & Rusdi 2020), further highlighting the interspecific variability in floral developmental patterns among orchids.

The floral spike, or inflorescence, is essential to an orchid’s reproductive anatomy. The stalk from which the individual blooms of an orchid species emerge is called the inflorescence. The orchid’s inflorescence is characterised by its high degree of specialisation and exhibits a wide range of appearances across various orchid genera and species. In species like *Phalaenopsis amabilis*, the floral spike emerges from the axillary buds at the fourth node below the apical leaf. The other axillary buds, however, stay dormant for the entire flowering season (Sakanishi *et al*. 1980). Mohamad and Rusdi (2020) observed that the inflorescence of *Renanthera bella* originates from the interstitial leaf and gradually grows and extends to create a flower bud in an acropetal chain. The other results suggested that the initial growth of inflorescence occurs sequentially (Harris *et al*. 1991; Naghiloo & Classen-Bockhoff 2017).

Floret primordia, also called flower primordia, are small, underdeveloped structures that eventually grow into individual blooms within orchid plants’ inflorescence (floral spike). The formation of floret primordia in orchids differs from that of other plants in several ways. The primordia are crucial elements in the orchid’s reproductive growth and signify the initial phases in creating blooms (Abad *et al*. 2017). Floret primordia will begin to differentiate at the axils of these bracts, causing the florets to advance into the pollen-formation stage (Wang *et al*. 2019). The development and growth of the pedicle, which supports the flower bud, may be observed two days following the emergence of the bud from the bract. Based on previous research on *Oncidium boissiense* by Tanaka *et al*. (1986), they categorised the floret development stages into seven different stages: (i) floret primordium formation, (ii) sepal formation, (iii) labellum formation, (iv) petal formation, (v) column formation, (vi) stigma and anther formation, and (vii) pollen formation.

Every full bloom of the flower is positioned on a little stem known as a pedicel (Sailo *et al*. 2019). The flower’s width measures 3.24 ± 0.11 cm, which closely aligns with the findings given by Paraste *et al*. (2023). The column is the stem structure of the orchid flower, and within its apical region is a pollinarium, an anther holding pollen (Paraste *et al*. 2023). Unlike most angiosperms that produce loose pollen grains, orchids make a compact structure called pollinia, which contains pollen. The pollinia structure facilitates the deposition of substantial amounts of pollen on the stigma, enabling the fertilisation of numerous ovules. The labellum of *A. odorata* has extended side lobes that extend towards the anther cap but do not reach up to the column. Certain orchids exhibit a lip that extends to the column, creating a hood-like structure that conceals the column (Teoh 2021). The lip’s edge is serrated, and the spurs are indented.

The flowering of *A. odorata* lasts 2 weeks in the absence of pollination, consistent with Teoh’s (2021) observation, after which it senesces. In addition, the result of this observation is also similar to other reports on the same genus, which is *Aerides multiflorum*, where rapid senescence is also observed in their pollinated flower (Attri *et al*. 2020). These observations align with *Phalaenopsis* and *Cymbidium* species, whose flowers, if unpollinated, were shown to live up to 8 weeks before dying within 7 days of pollination. In *Paphiopedilum*, unpollinated flowers exhibited longevity of up to three months, whereas pollinated flowers underwent accelerated senescence, wilting within approximately three weeks (Arditti 1992). In the case of *C. pendulum*, *C. aloifolium* and *Rhynchostylus retusa*, the flower only stands for 6 to 8 days after pollination, which is unlike 16 to 20 days when unpollinated (Attri *et al*. 2007; 2008a). According to Barthlott *et al*. (2014), the seed capsule can be categorised into five groups based on seed length. The phenomenon of floral senescence exhibits significant differences across different species. In particular species, the petals gradually lose their vitality and may eventually detach from the flower. In other species, the perianth undergoes abscission while still fully hydrated. In other species, the onset of the aging process is indicated by a colour change in either the entire perianth or a portion of it (Stead & Van Doorn 2010).

A different flower will have a different effect after pollination (Yam *et al*. 2009). Fertilisation occurs when a pollen grain is deposited onto the stigma of a female parent. The ovary undergoes an expanded size and maturation, resulting in the formation of seed pods. A single fertilisation event may produce hundreds of thousands of seeds (Benzing 1985). In this study, flowers of *A. odorata* were subjected to hand pollination one week after anthesis. At this stage, flowers were completely open and physiologically functional, indicating readiness for pollination. It is advisable to pollinate young, completely open flowers since these flowers exhibit the highest level of pollen receptivity. The time ranges from one to eight days after the blooming of flowers (Proctor 1998, Shiau *et al*. 2002). Furthermore, the utilisation of young flowers, which are less than a week old, ensures the receptivity of the stigmatic surface towards pollen. After two weeks, flowers undergo a process of closure, transforming pollen into a brown colour and making it unresponsive (Sivanaswari *et al*. 2011).

The development of an immature seed capsule or pod of *A. odorata* occurs 7 days following pollination. Pollen germination commenced within 24 h–48 h after pollination, coinciding with the lip colour change (Attri *et al*. 2020). Some orchid species even change colour 1 day after pollination (DAP) (Attri *et al*. 2007; 2008b; Attri & Nayyar 2021). The pollination serves as a stimulus for growth, leading to an increase in both the size of the column and the ovary within 24 hours following pollination. This action is commonly observed in most epidendroid orchids, where the ovule develops after pollination (Zhang & O’Neill 1993). However, different types of orchids may have different numbers of slits; some may have 3 to 6 slits, depending on the seed capsule structure (Wang *et al*. 2019). Pollen germination is initiated within 24 h to 48 h following pollination, which correlates with a lip colour modification (Attri *et al*. 2020).

Floral initiation and development are essential for plant reproduction because they mark the transition from vegetative growth to reproductive growth, allowing plants to produce flowers and seeds. Floral initiation is influenced by various environmental factors, including light intensity, temperature and nutrients (Gol *et al*. 2017; Wang *et al*. 2020; Guo *et al*. 2022; Nelson *et al*. 2024). It is a significant phase change in plants, where developmental programs switch from vegetative to reproductive growth (Lebedeva *et al*. 2020). The timing of floral initiation is crucial for determining flowering time and yield in crop plants (Zhang 2016). The regulation of floral initiation involves complex molecular mechanisms, including the involvement of specific genes and proteins. Understanding the regulation of floral initiation and development is important for optimising plant growth and reproduction and for developing management strategies in greenhouse and agricultural production. Orchid flower development is influenced by environmental signals and endogenous developmental programs controlled by genetic factors (Kaur *et al*. 2022). The understanding of the molecular mechanisms underlying orchid floral transition and flower development has been widened through advances in molecular and genetic technologies (Khadr *et al*. 2020).

## CONCLUSION

This work has effectively documented the initiation of the flowering pattern of *A. odorata* flowers and determined the development pattern for their floral organs. The study revealed that it took approximately two months for this species to develop its entire set of inflorescences completely. Even though daily observations were conducted rigorously for two months, the complete floral-to-capsule progression was recorded for roughly 110 days, guaranteeing that the flowering phase and capsule maturation were included in this research. The flower’s development can be viewed via 12 distinct stages, each taking over a week to complete. According to the collected data, the optimal time to pollinate is on the fourth and fifth day after the flower has fully bloomed. The ideal timeframe for harvesting the seed capsule is between 100 days and 110 days. Hence, the results of this investigation could assist in identifying the perfect age of capsules for the efficient and prosperous in vitro multiplication of this endangered orchid species. Nevertheless, it is important to emphasise that the current findings are based on observations of three flowering of *A. odorata* with multiple inflorescences, thus offering foundational descriptive data instead of generalisations applicable to the entire species. For future studies, it is advisable to utilise larger sample sizes and broader replication to affirm and elaborate on these results while incorporating molecular and genetic methods that could further elucidate the regulatory mechanisms that control floral initiation and capsule development in *A. odorata*.

## Figures and Tables

**TABLE 1:** The floral development stages of *A. odorata* Lour.

Stages	Landmark events/ development sequences	Duration (days)	Description of events
1	Spike formation starts to swell	5–7	The bud is enlarged and exhibits a pale green in contrast to the flower’s stalk.
2	Development of flower bud primordia	24	The floral bud transforms, becoming more irregular in shape and elongated in structure.
3	Initiation development of the flower bud	32	The process of bud formation is more distinct, with the emergence of several initial bud shapes.
4	Formation of an immature flower bud	37	The peduncle undergoes elongation, resulting in the formation of interspaces between individual buds.
5	Formation of a mature flower bud	52	The bud underwent maturation and developed into a fully grown flower, complete with the labellum structure. The outer surface of the bud displays a blend of purplish and pale green.
6	Flower buds start to open partially	57	The petals and sepals initiate the process of unfolding, exhibiting a pale pink hue on both anatomical components. The labellum exhibits a green colour.
7	Fully bloomed/ flowering of *A. odorata*	67	The petals and sepals exhibit a pink colour, accompanied by a purple tinge at the periphery of each structure. The labellum’s pouch is reddish, while the remainder of the structure is yellow.
8	Initiation of flower senescence	79	The flower begins to change into a bright colour in all structures three days after undergoing hand pollination.
9	Flower senescence	84	The flower is fully wilted 84 days after being pollinated.
10	Formation of an immature capsule	90	Capsules are light green to yellowish.
11	Formation of a mature capsule	100	The mature capsule appeared to be green to brownish.
12	Capsule senescence	110	The capsule split and opened.

## References

[b1-tlsr_37-1-133] Abad U, Sassi M, Traas J (2017). Flower development: From morphodynamics to morphomechanics. Philosophical Transaction of The Royal Society of London. Series B, Biological Sciences.

[b2-tlsr_37-1-133] Arditti J (1992). Fundamentals of orchid biology.

[b3-tlsr_37-1-133] Attri LK, Nayyar H (2021). Pollination related temporal sequences of reproductive development and tracing the path of pollen tubes in *Cymbidium pendulum* (Roxb.) Sw., an ornamental orchid. Flora.

[b4-tlsr_37-1-133] Attri LK, Nayyar H, Bhanwra RK (2020). Pollination induced embryological studies in *Aerides multiflora* (Roxb). International Journal of Botany Studies.

[b5-tlsr_37-1-133] Attri LK, Nayyar H, Bhanwra RK, Pehwal A (2008a). Pollination-induced oxidative stress in floral organs of *Cymbidium pendulum* (Roxb.) Sw. and *Cymbidium aloifolium* (L.) Sw. (Orchidaceae): A biochemical investigation. Scientia Horticultureae.

[b6-tlsr_37-1-133] Attri LK, Nayyar H, Bhanwra RK, Pehwal A (2008b). Post-pollination changes in the floral organs of two Cymbidium species. Biologia Plantarum.

[b7-tlsr_37-1-133] Attri LK, Nayyar H, Bhanwra RK, Vij SP (2007). Post-pollination biochemical changes in the floral organs of *Rhynchostylis retusa* (L.) Bl. and *Aerides multiflora* Roxb. (Orchidaceae). Journal of Plant Biology.

[b8-tlsr_37-1-133] Bakar B, Latip MA, Gansau JA (2014). Asymbiotic germination and seedling development of *Dimorphics lowii* (Orchidaceae). Asian Journal of Plant Science.

[b9-tlsr_37-1-133] Balilashaki K, Gantait S, Naderi R, Vahedi M (2015). Capsule formation and asymbiotic seed germination in some hybrids of Phalaenopsis, influenced by pollination season and capsule maturity. Physiology and Molecular Biology of Plants.

[b10-tlsr_37-1-133] Barthlott W, Grosse-veldmann B, Korotkova N (2014). Orchid seed diversity: A scanning electron microscopy survey. Englera.

[b11-tlsr_37-1-133] Benzing DH (1985). Vascular epiphytism: Taxonomic participation and adaptive diversity. Annals of the Missouri Botanical Garden.

[b12-tlsr_37-1-133] Chen XQ, Wood JJ, Wu ZG,  Raven PH, Hong DY (2009). Aerodes loureiro FI. Cochinch. Flora in China. Orchidacae.

[b13-tlsr_37-1-133] Gol L, Tomé F, Von Korff M (2017). Floral transitions in wheat and barley: Interactions between photoperiod, abiotic stresses, and nutrient status. Journal of Experimental Botany.

[b14-tlsr_37-1-133] Guo H, Zhing Q, Tian F, Zhou X, Tan X, Luo Z (2022). Transcriptome analysis reveals putative induction of floral initiation by old leaves in tea-oil tree (*Camellia oleifera* ‘changlin53’). International Journal of Molecular Sciences.

[b15-tlsr_37-1-133] Gurung DB (2006). An illustrated guide to the orchids of Bhutan.

[b16-tlsr_37-1-133] Harris EM, Tucker S, Urbatsch L (1991). Floral initiation and early development in *Erigeron philadelphicus* (Asteraceae). American Journal of Botany.

[b17-tlsr_37-1-133] Iwamoto A, Bull-Hereñu K (2018). Floral development: Re-evaluation of its importance. Journal of Plant Research.

[b18-tlsr_37-1-133] Kaur H, Kumar A, Choudhary A, Parmar H, Rashid A, Mehta S, Husen A, Husen A (2022). Metabolism during adventitious root primordia initiation and development. Environmental, physiological and chemical controls of adventitious rooting in cuttings.

[b19-tlsr_37-1-133] Kauth PJ, Johnson TR, Stewart SL, Kane ME (2008). A classroom exercise in hand pollination and in vitro asymbiotic orchid seed germination. Plant Cell Tissue and Organ Culture.

[b20-tlsr_37-1-133] Khadr A, Wang G-L, Wang Y-H, Zhang R-R, Wang X-R, Xu Z-S, Tian Y-S, Xiong A-S (2020). Effects of auxin (indole-3-butyric acid) on growth characteristics, lignification, and expression profiles of genes involved in lignin biosynthesis in carrot taproot. PeerJ.

[b21-tlsr_37-1-133] Khalida A, Suwirman, Noli AZ (2019). Induksi kalus Anggrek lilin (*Aerides odorata* Lour.) dengan pemberian beberapa konsentrasi 2,4 diklorofenoksiasetat (2,4 D). Jurnal Biologi UNAND.

[b22-tlsr_37-1-133] Lebedeva MA, Dodueva IE, Gancheva MS, Tvorogova VE, Kuznetsovaka KA, Lutova LA (2020). The evolutionary aspects of flowering control: Florigens and anti-Florigens. Russian Journal of Genetics.

[b23-tlsr_37-1-133] Li Y, Zhang B, Yu H (2022). Molecular genetic insights into orchid reproductive development. Journal of Experimental Botany.

[b24-tlsr_37-1-133] Mohamad NN, Rusdi NA (2020). Scanning electron microscopy analysis of early floral development in *Renanthera Bella* J. J. Wood, an endemic orchid from Sabah. Pertanika Journal of Tropical Agriculture and Science.

[b25-tlsr_37-1-133] Naghiloo S, Classen-Bockhoff R (2017). Understanding the unique flowering sequence in *Dipsacus fullonum*: Evidence from geometrical changes during head development. PLOS One.

[b26-tlsr_37-1-133] Nelson HV, Gansau JA, Mus AA, Mohammad NN, Shamsudin NA, Amin J, Rusdi NA (2023). Developing *Paraphalaenopsis labukensis* (Shim, A. Lamb & C.L. Chan), an orchid endemic to Sabah, Borneo, asymbiotic seed germination and in vitro seedling development. Horticulturea.

[b27-tlsr_37-1-133] Nelson HV, Gansau JA, Shamsudin NA, Rusdi NA (2024). Flower initiation pattern, developmental stages, and seed morphology of *Paraphalaenopsis labukensis* P.S. Shim, A. Lamb & C.L. Chan, an endangered Orchid in Sabah. The Open Agriculture Journal.

[b28-tlsr_37-1-133] Nilsson LA (1992). Orchid pollination biology. Trends in Ecology & Evolution.

[b29-tlsr_37-1-133] Owens JN, Sornsathapornkul P, Tangmitchareon S (1996). Studying flowering and seed ontogeny in tropical forest trees.

[b30-tlsr_37-1-133] Paraste VK, Sarsaiya S, Mishra UC, Sourabh P (2023). A comprehensive review on global research trends on *Aerides* genus with reference to *Aerides odorata* species. Journal of Applied Biology and Biotechnology.

[b31-tlsr_37-1-133] Proctor HC (1998). Effect of pollen age on fruit set, fruit weight, and seed set in three orchid species. Canadian Journal of Botany.

[b32-tlsr_37-1-133] Sailo N, Rai D, De LC (2019). Physiology of temperate and tropical orchids: An overview. International Journal of Scientific Research.

[b33-tlsr_37-1-133] Sakanishi Y, Imanishi H, Ishida G (1980). Effect of temperature on growth and flowering of *Phalaenopsis amabilis*. Bulletin of the University of Osaka Prefecture B.

[b34-tlsr_37-1-133] Shiau YJ, Sagare AP, Chen UC, Yang SR, Tsay HS (2002). Conservation of *Anoectochilus formosanus* Hayata by artificial cross-pollination and in vitro culture of seeds. Botanical Bulletin of Academia Sinica.

[b35-tlsr_37-1-133] Singh D, Sharma S, Jose-Santhi J, Kalia D, Singh RK (2023). Hormones regulate the flowering process in saffron differently depending on the developmental stage. Frontier Plant Science.

[b36-tlsr_37-1-133] Sivanaswari C, Thohirah LA, Fadelah AA, Abdullah NAP (2011). Hybridization of several Aerides species and in vitro germination of its hybrid. African Journal of Biotechnology.

[b37-tlsr_37-1-133] Stead AD, Van Doorn WG, Scott RJ, Stead AD (2010). Strategies of flower senescence: A review. Molecular and cellular aspects of plant reproduction.

[b38-tlsr_37-1-133] Sulistiarini D (2008). Keanekaragaman jenis anggrek Pulau Wawonii. Berkala Penelitian Hayati.

[b39-tlsr_37-1-133] Syamsuwida D, Palupi ER, Siregar IZ, Indrawan A (2012). Flower initiation, morphology, and developmental stages of flowering-fruiting of mindi (*Melia azedarach* L.). Jurnal Manajemen Hutan Tropika.

[b40-tlsr_37-1-133] Tanaka M, Shinya Y, Goi M (1986). Morphological observation on vegetative growth and flower bud formation in *Oncidium boissiense.*. Scientia Horticulturea.

[b41-tlsr_37-1-133] Teoh ES (2016). Medicinal orchids of Asia.

[b42-tlsr_37-1-133] Teoh ES (2021). Orchid species from Himalaya and Southeast Asia (A–E).

[b43-tlsr_37-1-133] Wang R, Gui Y, Zhao T, Ishii M, Eguchi M, Xu H, LIT, Iwasaki Y (2020). Determining the relationship between floral initiation and source-sink dynamics of tomato seedlings affected by changes in shading and nutrients. HortScience: American Society for Horticultural Science.

[b44-tlsr_37-1-133] Wang SL, Viswanath KK, Tong CG, An HR, Jang S, Chen FC (2019). Floral induction and flower development of Orchids. Frontiers in Plant Science.

[b45-tlsr_37-1-133] Yam T, Cheng W, Neng Y, Avadhani PN, Hew CS, Arditti J, Kurzweil H, Kull T, Arditti J, Wong SM (2009). History-physiology: Pollination effects on orchid flowers and the first suggestion by Professor Hans Fitting 1877–1970 that plants produce hormones. Orchid biology: Reviews and perspectives, X.

[b46-tlsr_37-1-133] Yoshida A, Taoka K, Hosaka A, Tanaka K, Kobayashi H, Muranaka T, Toyooka K, Oyama T, Tsuj H (2021). Characterization of frond and flower development and identification of FT and FD genes from Duckweed *Lemna aequinoctialis*. Frontiers in Plant Science.

[b47-tlsr_37-1-133] Zhang D (2016). Plant development and reproduction. Chinese Science Bulletin.

[b48-tlsr_37-1-133] Zhang XS, O’Neill SD (1993). Ovary and gametophyte development are coordinately regulated by auxin and ethylene following pollination. Plant Cell.

